# Epigenetic Modulation of Class-Switch DNA Recombination to IgA by miR-146a Through Downregulation of Smad2, Smad3 and Smad4

**DOI:** 10.3389/fimmu.2021.761450

**Published:** 2021-11-16

**Authors:** Paolo Casali, Shili Li, Grecia Morales, Cassidy C. Daw, Daniel P. Chupp, Amanda D. Fisher, Hong Zan

**Affiliations:** Department of Microbiology, Immunology & Molecular Genetics, University of Texas Long School of Medicine, UT Health Science Center, San Antonio, TX, United States

**Keywords:** AID, B cell, class switch DNA recombination (CSR), epigenetics, IgA, miR-146a, microRNA, Smad2/Smad3/Smad4

## Abstract

IgA is the predominant antibody isotype at intestinal mucosae, where it plays a critical role in homeostasis and provides a first line of immune protection. Dysregulation of IgA production, however, can contribute to immunopathology, particularly in kidneys in which IgA deposition can cause nephropathy. Class-switch DNA recombination (CSR) to IgA is directed by TGF-β signaling, which activates Smad2 and Smad3. Activated Smad2/Smad3 dimers are recruited together with Smad4 to the *IgH* α locus *Iα* promoter to activate germline Iα-Cα transcription, the first step in the unfolding of CSR to IgA. Epigenetic factors, such as non-coding RNAs, particularly microRNAs, have been shown to regulate T cells, dendritic cells and other immune elements, as well as modulate the antibody response, including CSR, in a B cell-intrinsic fashion. Here we showed that the most abundant miRNA in resting B cells, miR-146a targets *Smad2, Smad3* and *Smad4* mRNA 3’UTRs and keeps CSR to IgA in check in resting B cells. Indeed, enforced miR-146a expression in B cells aborted induction of IgA CSR by decreasing Smad levels. By contrast, upon induction of CSR to IgA, as directed by TGF-β, B cells downregulated miR-146a, thereby reversing the silencing of *Smad2, Smad3* and *Smad4*, which, once expressed, led to recruitment of Smad2, Smad3 and Smad4 to the Iα promoter for activation of germline *Iα-Cα* transcription. Deletion of miR-146a in *miR-146a* ^–/–^ mice significantly increased circulating levels of steady state total IgA, but not IgM, IgG or IgE, and heightened the specific IgA antibody response to OVA. In *miR-146a* ^–/–^ mice, the elevated systemic IgA levels were associated with increased IgA^+^ B cells in intestinal mucosae, increased amounts of fecal free and bacteria-bound IgA as well as kidney IgA deposition, a hallmark of IgA nephropathy. Increased germline *Iα-Cα* transcription and CSR to IgA in *miR-146a* ^–/–^ B cells *in vitro* proved that miR-146a-induced Smad2, Smad3 and Smad4 repression is B cell intrinsic. The B cell-intrinsic role of miR-146a in the modulation of CSR to IgA was formally confirmed *in vivo* by construction and OVA immunization of mixed bone marrow *μMT/miR-146a* ^–/–^ chimeric mice. Thus, by inhibiting *Smad2*, *Smad3* and *Smad4* expression, miR-146a plays an important and B cell intrinsic role in modulation of CSR to IgA and the IgA antibody response.

## Introduction

IgA is the predominant antibody isotype at mucosal surfaces and the most abundant immunoglobulin (Ig) isotype in the body (1–5). It plays a critical role in mucosal homeostasis in the gastrointestinal, respiratory and urogenital tracts. IgA also provides a first line of immune protection against harmful commensals as well as airborne, ingested and sexually transmitted pathogens that come into contact with such mucosal surfaces (6). In the gut, IgA dimers are selectively transported across epithelia into the intestinal lumen, where they neutralize toxins and block the entry of pathogenic bacteria and viruses across the intestinal epithelium (4, 7). Gut IgA effect both immune protection and immune exclusion in a non-inflammatory manner, thereby playing a central role in host-microbial interaction. Intestinal IgA comprise low-affinity antibodies, mostly polyreactive, that prevent commensal bacteria from penetrating the mucosal barrier, as well as high-affinity IgA that neutralize toxins and microbial pathogens (8). Low affinity IgA would be generated in a T cell-independent fashion, generally in the lamina propria, while high affinity IgA would be generated in a T cell-dependent way, mainly in germinal centers of Peyer’s patches.

Whether in T cell-dependent or independent fashion, IgA are produced through class-switch DNA recombination (CSR), a process that requires germline transcription of Ig heavy chain constant α gene (*Igα*), as activated by induction of the *Iα* promoter, and expression of activation-induced cytidine deaminase (AID, encoded by *Aicda* gene) (8–11). Like CSR to other isotypes, CSR to IgA is a tightly controlled process. It is initiated by TLR ligand:TLR or CD154:CD40 engagement and TGF-β engagement of the TGF-β receptor. The latter activates (by phosphorylation) Smad2 and Smad3 transcription factors (12, 13). Activated Smad2/Smad3 heterodimers form complexes with Smad4 for nuclear translocation and docking onto Smad-binding elements (SBEs) in the *Iα* promoter (8, 14–16). Here, in conjunction with co-factors, such as Runx3 and Pu.1, Smad2, Smad3 and Smad4 activate germline *Iα-Cα* transcription, which critically initiates CSR to IgA (17–20). Accordingly, overexpression of Smad3 and Smad4 in B cells increased IgA expression (15) and B cell-specific Smad2 deletion reduced CSR to IgA (20). Other cytokines, such as IL-4 and IL-5, can enhance CSR to IgA, generally by boosting secretion of endogenous B cell TGF-β (21). Retinoic acid (RA), a vitamin A metabolite that is abundant in the gut, has been shown to induce gut homing receptors on activated T cells and CSR to IgA throughout gut-associated lymphoid tissue (GALT). Indeed, impaired GALT dendritic cells conversion of retinol to RA leads to decreased intestinal IgA, resulting in a noticeably decreased IgA in the circulating blood (22).

Epigenetic factors and modifications, such as non-coding RNAs, including microRNAs (miRNAs) and long non-coding RNAs (lncRNAs), DNA methylation and histone post-translational modifications effect heritable changes in gene expression that are independent from genomic DNA sequence. As we have shown, epigenetic factors and modifications act in concert with transcription factors to regulate B cell CSR, somatic hypermutation (SHM), plasma cell differentiation and generation of memory B cells, thereby shaping the antibody response to foreign- and self-antigens (11, 23–27). In particular, select miRNAs silence B cell *Aicda* and *Prdm1* genes, thereby downregulating AID enzyme and Blimp1 transcription factor, which critically mediate CSR/SHM and plasma cell differentiation (11, 23, 25, 28–30). Prior to modulating these peripheral B cell differentiation processes, miRNAs regulate B development by modulating the expression of genes at the pro-B and pre-B cell stages (31, 32).

miR-146a is one of a small number of miRNAs, whose cell expression is strongly induced by challenge with bacterial endotoxins, and whose prolonged expression has been linked to immune tolerance, implying that it acts as a fine-tuning mechanism to prevent an overstimulation of the immune system and, possibly, inflammatory response (33–35). Indeed, miR-146a has been suggested to regulate immune cells, such as T cell, dendritic cells and macrophages, as well as myeloid cells by exerting immunosuppressive and anti-inflammatory functions (36–42). These would include suppression of gut immunity, autoimmunity and myeloproliferation (43–45). miR-146a is one of the most abundant miRNAs in B cells, particularly resting B cells (38, 44), and its role in B cell function just starts getting attention. miR-146a has been shown to control germinal center responses by targeting multiple components of the CD40 signaling pathway in B cells (39). miR-146a expression has been suggested to reduce IgA production in the gut, and miR-146a deficiency has led to development of immune complex glomerulonephritis in mice (45, 46). miR-146a G/C polymorphism rs2910164 has been shown to associate with pediatric hyper IgA and IgA nephropathy, a syndrome that is characterized by high serum IgA levels, and IgA deposition in glomeruli, as well as with diabetic nephropathy (47, 48). Such a miR-146a G/C polymorphism seemingly reduced the expression level of pre-miR-146a, likely by affecting the binding efficiency of the Drosha/DGCR8 complex to pri-miR-146a, thereby eventually reducing the amount of mature miR-146a (49). Further, miR-146a was found to act as a negative regulator of TGF-β signaling in skeletal muscle cells (50). Finally, it was shown to target Smad4, as induced by the TGF-β signaling pathway in promyelocytic leukemia cells (51).

The above findings and considerations led us to hypothesize that miR-146a plays an important role in regulating B cell CSR to IgA by modulating TGF-β-induced Smad2, Smad3 and Smad4. We tested our hypothesis by analyzing *miR-146a* ^–/–^ mice for basal level of circulating IgA as well as frequency of IgA^+^ B cells in spleen, bone marrow, mesenteric lymph nodes (MLNs), lamina propria and Peyer’s patches, fecal bacteria-bound and free IgA as well as IgA deposition in kidney glomeruli. We further analyzed the magnitude of a specific IgA antibody response in *miR-146a* ^-/-^ mice as well as constructed *μMT/miR-146a* ^–/–^ mixed bone marrow chimera mice to prove that any miR-146a related dysregulation in IgA production was due to intrinsic B cell miR-146a. We also tested the *miR-146a* ^–/–^ B cell intrinsic ability to undergo CSR to IgA *in vitro* in response to T-dependent and T-independent CSR-inducing stimuli. In these *in vitro* CSR-induced B cells, we measured the expression of *Smad2, Smad3, Smad4* transcripts, the recruitment of Smad2, Smad3 and Smad4 transcription factors to the Iα promoter and consequent germline *Iα-Cα* transcription. Finally, we enforced expressed miR-146 in B cells to analyze the impact of this miRNA on Smad2, Samd3 and Smad4 expression levels. We concluded that miR-146a plays an important role in keeping in check *Smad2, Smad3* and *Smad4* transcription in resting B cells, and inhibiting CSR to IgA. Upon exposure to IgA CSR-inducing stimuli, such as CD154 or LPS plus TGF-β, B cells physiologically downregulate miR-146a, thereby allowing for increased Smad2, Smad3 and Smad4 expression, germline *Iα-Cα* transcription and unfolding of IgA CSR.

## Materials and Methods

### Mice

C57BL/6 (CD45.2^+^, stock No. 000664), C57/CD45.1^+^ (B6.SJL-Ptprc^a^Pepc^b^/BoyJ, stock No. 002014), *miR-146a^–/–^* (B6.Cg-*Mir146^tm1.1Bal^*/J, CD45.2^+^, stock No. 016239) (52) and *μMT* (B6.129S2-*Ighm^tm1Cgn^*/J, CD45.2^+^, stock No. 002288) (53) mice were purchased from the Jackson Laboratory. miR*-146a^–/–^* and *miR-146a^+/+^* mice (8-12 wk of age) were analyzed for fecal free and bacteria-bound IgA using our published procedure (23). They were also analyzed for spleen, bone marrow, MLNs, lamina propria and Peyer’s patches IgA^+^ B cells using an anti-mouse IgA mAb (clone C10-3, BD Bioscience) and mAb to mouse CD19 (clone 1D3, BD Bioscience). All mice were housed and maintained in the University of Texas Health Science Center San Antonio pathogen–free vivarium and provided with autoclaved food and deionized water. The Institutional Animal Care and Use Committees (IACUC) of the University of Texas Health San Antonio approved all animal research protocols.

To construct mixed bone marrow chimeric B cell-specific *miR-146a^–/–^* (*μMT/miR-146a^–/–^*) mice and controls *μMT/miR-146a^+/+^* mice, sex-matched recipient C57/CD45.1^+^ mice (8-12 wks of age) from the same breeding batch were treated with neomycin sulfate (2 mg/ml in drinking water) for one week before being γ-irradiated for complete myeloablation (1000 Rad or 10 Gy, 12.5 min from a ^137^cesium source). After 24 h, the mice were randomized into two groups and injected through the tail vein with 2.5 x 10^6^ mixed bone marrow cells as comprised of 80% (2 x 10^6^) cells from a *μMT* mouse and 20% (0.5 x 10^6^) cells from either a *miR-146a^–/–^* mouse or its *miR-146a^+/+^* littermate. Prior to mixing, bone marrow cells isolated form tibia and fibula of donor *miR-146a^–/–^* or *miR-146a^+/+^* mice were depleted of T cells by incubation with biotinylated anti-CD3 mAb (Magnisort^®^ Streptavidin Negative Selection Beads (eBioscience). Chimeric mice were monitored by flow cytometry for CD45.1 (clone A20, 110471, BioLegend), CD45.2 (clone 104, 109830, BioLegend), CD19 (clone 1D3, 20-0193, Tonbo), CD4 (clone RM4-5, 45-0042-82, eBioscience), CD8 (clone H35-17.2, 12-0083-81, eBioscience), CD11b (clone M1/70, 101212, BioLegend), and/or CD11c (clone N418, 117323, BioLegend) expression on circulating mononuclear cells for at least 6 wks to confirm the immune system reconstitution.

For antigen-specific IgA response experiments, miR*-146a^–/–^* and *miR-146a^+/+^* mice as well as *μMT*/*miR-146a^–/–^* and *μMT*/*miR-146a^+/+^* mice were given ovalbumin (OVA, 20 mg) with cholera toxin (OVA CT) *via* intragastric gavage (23) at d 0, 7 and 14. Sera and feces were collected at d 21 (for total and OVA-specific IgM, IgG1, IgA and IgE analysis), at which time mice were sacrificed for all other studies.

### ELISAs and ELISPOTs

Titers of IgG1 and IgA in cell culture supernatants of *in vitro*-stimulated B cells or IgM, IgG1, IgA or IgE *in vivo* titers of circulating/fecal total and/or OVA-specific-IgM, IgG1, IgA and IgE were measured using specific ELISAs, as we described (28, 54–56). For antibody secreting cell (ASC) analysis, MultiScreen^®^ ELISPOT plates (MAIPS4510; Millipore) were activated with ethanol (35%), washed four times with PBS and coated with unlabeled rabbit polyclonal antibodies against mouse IgM, IgG1 or IgA in PBS overnight at 4°C. The plates were then washed six times with PBS, blocked with BSA (0.5%) in RPMI/HEPES plus L-glutamine for 1 h at room temperature. The plates were then used to culture single cell suspensions from MLNs, Peyer’s patches, bone marrow and spleen cells at 37°C for 16 h in 10% FBS RPMI medium containing 50 mM 2-ME and 1× antibiotic-antimycotic mixture (15240-062; Invitrogen) (FBS-RPMI) at 150,000 cells/ml. The culture fluids were then removed, the plates were washed six times, incubated with biotin-anti-IgM, IgG1, or IgA Ab for 2 h on a shaker at room temperature, washed, incubated with horseradish peroxidase (HRP)-streptavidin (Santa Cruz Biotech) for 1 h on a shaker at room temperature, washed again and developed using the Vectastain AEC peroxidase substrate kit (SK-4200, Vector Laboratories) following manufacturer’s protocol. ASCs were imaged and quantified using a CTL-ImmunoSpot Analyzer and software.

### B Cells, CSR and Plasma Cell Differentiation

Naive IgM^+^IgD^+^ B cells were isolated from 8- to 12-wk-old C57BL/6 mice, as described (9). B cells were resuspended in FBS-RPMI at 37°C in 48-well plates and stimulated with LPS (5 μg/ml) from *Escherichia coli* (055:B5; Sigma-Aldrich) for CSR to IgG3; LPS (3 μg/ml) or CD154 [1 U/ml; obtained from membrane fragments of baculovirus-infected Sf21 insect cells (45)] plus IL-4 (5 ng/ml; R&D Systems) for CSR to IgG1/IgE and plasma cell differentiation; or LPS (3 μg/ml) or CD154 (1 U/ml) plus TGF-β (2 ng/ml; R&D Systems), IL-4 (5 ng/ml), IL-5 (3 ng/ml; R&D Systems), RA (10 nM, Sigma) and anti-Igδ mAb for CSR to IgA. After 96 h, the cells were stained with FITC-labeled rat mAb to mouse IgG1 (clone A85-1), mouse IgG2a/c (clone R19-15), mouse IgG3 (clone R40-82), mouse IgA (clone C10-3), or PE-labeled rat mAb to mouse CD19 (clone 1D3), all from BD Biosciences, 7-Aminoactinomycin D (7-AAD; A1310, Invitrogen) and PE-Cy7-anti-CD138 mAb (142513, Biolegend). The same mAbs were used to stain mouse mononuclear cells *ex vivo*. Cell analyses were performed using a LSR-II flow cytometer (BD Biosciences), and data were analyzed using FlowJo software (TreeStar). All experiments were performed in triplicates.

### Quantitative RT-PCR (qRT-PCR) of mRNAs and miRNAs

For quantification of germline *I_H_-C_H_*, post-recombination *Iμ-Cα* and circular *Iα-Cμ* transcripts, as well as *Smad2, Smad3, Smad4*, *Aicda, Prdm1, Traf6* and *Irak1* transcripts, RNA was extracted from 0.2-5.0 × 10^6^ cells using either Trizol^®^ Reagent (Invitrogen) or RNeasy Plus Mini Kit (Qiagen). Residual DNA was removed from using gDNA eliminator columns (Qiagen). cDNA was synthesized from total RNA with the SuperScript™ III First-Strand Synthesis System (Invitrogen) using oligo-dT primer. Transcript expression was measured by qRT-PCR with the appropriate primers (**Supplementary Table 1**) using a Bio-Rad MyiQ™ Real-Time PCR Detection System (Bio-Rad Laboratories) to measure SYBR Green (IQ™ SYBR^®^ Green Supermix, Bio-Rad Laboratories) incorporation with the following protocol: 95°C for 15. sec, 40 cycles of 94°C for 10 sec, 60°C for 30 sec, 72°C for 30 sec. Data acquisition was performed during 72°C extension step. Melting curve analysis was performed from 72-95°C.

For quantification of mature miRNA transcripts, RNA was extracted from 0.2-5.0 × 10^6^ cells using miRNeasy^®^ Mini Kit (Qiagen) and reverse-transcribed with miScript II RT Kit (Qiagen) using miScript HiSpec buffer. A Bio-Rad MyiQ™ Real-Time PCR Detection System was used to measure SYBR Green (miScript SYBR Green PCR Kit; Qiagen) incorporation according to manufacturer’s instructions. Mature miRNA forward primers (**Supplementary Table 1**) were used at 250 nM in conjunction with the Qiagen miScript Universal Primer and normalized to expression of small nuclear/nucleolar RNAs Rnu6/RNU61/2, Snord61/SNORD61, Snord68/SNORD68, and Snord70/SNORD70. The ΔΔCt method was used for miRNA qRT-PCR data analysis.

### Fluorescence Microscopy

To analyze IgM and IgA-producing cells in the lamina propria, the intestine was folded into a “Swiss-roll”, fixed with PFA (4%), and embedded in paraffin. Ten μm sections were cut and heated at 80°C to adhere to the slide, washed four times in xylene for 2 min, dehydrated two times with 100% ethanol for 1 min, two times with 95% ethanol for 1 min and washed two times in water for 1 min. Antigens were unmasked using 2 mM EDTA in 100°C for 40 mins followed by a cooling step at 25°C on the bench top, 3 times washing with 1x TBS and blocking using 10% BSA for 15 min. Slides were again washed three times with 1x TBS and stained with PE-anti-IgM, primary-rabbit anti mouse-IgA mAb (PA-1-30826, Thermo fisher) followed by Alexa Fluor 488^®^-anti rabbit-IgG (H+L) F(ab’)_2_ mAb (4412; Cell Signaling) for 2 h in a moist dark chamber. To analyze IgA-producing cells in MLNs, 10 μm MLN sections were prepared by cryostat and loaded onto positively charged slides, fixed in cold acetone and stained with rabbit anti mouse-IgA mAb (PA-1-30826, Thermo fisher) followed by Alexa Fluor 488^®^-anti rabbit-IgG (H+L) F(ab’)_2_ mAb (4412, Cell Signaling) for 1 h in a moist dark chamber. After washing three times with Triton X-100 (0.1%) in TBS, slides were air-dried and coverslips were mounted with ProLong^®^ Gold Antifade Reagent using DAPI (Invitrogen). Fluorescence images were captured using a 10 x objective lens with a Zeiss Axio Imager Z1 fluorescence microscope.

### Kidney Pathology

To analyze kidney IgA deposition, kidneys from miR*-146a^–/–^* and *miR-146a^+/+^* mice were fixed in 4% formaldehyde and paraffin-embedded for hematoxylin and eosin staining. For immunofluorescence analysis, 5-μm cryostat sections were loaded onto positively charged slides, fixed in cold acetone, and stained with FITC-labeled rat anti-mouse IgA mAb (clone C10-3, BD Biosciences). Cover slips were mounted using ProLong Gold Antifade Reagent for microscopy analysis.

### Microbiota Analysis

Microbial DNA were extracted from mouse feces using Quick-DNA™ Fecal/Soil Microbe Microprep Kit (Zymo Research) according to the manufacturer’s instructions. To determine the composition of the bacterial phyla present in mouse feces, isolated bacterial DNA was tagged and sequenced using miSeq platform. The V3-V4 hypervariable region of the bacteria 16S rRNA gene was amplified by PCR using tagged bact-341F primer 5’-TCGTCGGCAGCGTCAGATGTGTATAAGAGACAGCCTACGGGNGGCWGCAG-3’, bact-805R primer 5’-GTCTCGTGGGCTCGGAGATGTGTATAAGAGACAGGACTACHVGGGTATCTAATCC-3’ and Phusion DNA polymerase. Multiplexing indices and Illumina sequencing adapters were then added to the amplicons by limited-cycle amplification using the Nextera XT Index Kit (Illumina). The libraries were normalized, pooled and sequenced using the MiSeq system (Illumina). Sequencing and quality assessment were performed by the UT Health Genome Sequencing Facility. Bacterial taxonomy was assigned using the Ribosomal Database Project (RDP) classifier (57).

### Immunoblotting

After stimulation by CSR-inducing stimuli and culture, B cells were lysed in Laemmli buffer. Cell extracts containing equal amounts of protein (20 μg) were fractionated through SDS-PAGE (10%). The fractionated proteins were transferred onto polyvinylidene difluoride membranes (Bio-Rad Laboratories) overnight (30 V) at 4°C. After blocking and overnight incubation at 4°C with anti-Smad2 (A7699, Abclonal), anti-Smad3 (A19115, Abclonal) and anti-Smad4 (A5657, Abclonal) rabbit Abs, or anti-β-Actin mAb (AC-15, Sigma-Aldrich), the membranes were incubated with HRP-conjugated secondary Abs. After washing with PBS-Tween 20 (0.05%), bound HRP-conjugated Abs were revealed using Western Lightning^®^ Plus-Enhanced Chemiluminescence reagents (PerkinElmer Life and Analytical Sciences).

### Chromatin Immunoprecipitation (ChIP) Assays

ChIP assays were performed as described (23, 24, 30). Briefly, B cells (5 × 10^6^) were treated with 1% formaldehyde for 10 min at 25°C to cross-link chromatin. After washing with cold PBS containing protease inhibitors (Roche Applied Science), chromatin was separated using nuclear lysis buffer (10 mM Tris-HCl, 1.0 mM EDTA, 0.5 M NaCl, 1% Triton X-100, 0.5% sodium deoxycholate, 0.5% Sarkosyl, pH 8.0) and resuspended in IP-1 buffer (20 mM Tris-HCl, 200 mM NaCl, 2.0 mM EDTA, 0.1% sodium deoxycholate, 0.1% SDS, protease inhibitors). Chromatin was sonicated to yield ~0.2 – 1.0 kb DNA fragments, precleared with agarose beads bearing protein G (Santa Cruz Biotechnology), and then incubated with anti-Smad2 (A7699, Abclonal), anti-Smad3 (A19115, Abclonal) or anti-Smad4 (A5657, Abclonal) rabbit Abs at 4°C. After overnight incubation, immune complexes were isolated using agarose beads bearing protein G, eluted with elution buffer (50 mM Tris-HCl, 0.5% SDS, 200 mM NaCl, 100 μg/ml Proteinase K, pH 8.0), and then incubated at 65°C overnight to reverse formaldehyde cross-links. DNA was purified using QIAquick PCR Purification Kit (Qiagen). The purified DNA was used as a template for qPCR analysis of the *Iα* promoter using specific primers (**Supplemental Table 1**).

### Enforced Expression of miR-146a

The miR-146a expression retroviral construct pMSCV-PIG-miR-146a (58) was a gift from Joshua Mendell (Addgene plasmid # 64234) and the pMSCV-PIG (Puro IRES GFP) empty vector was a gift from David Bartel (Addgene plasmid # 21654). To generate the retrovirus, pMSCV-PIG-miR-146a or empty pMSCV-PIG vector were used together with the pCL-Eco retrovirus-packaging vector (Imgenex) to transfect HEK293T cells by the calcium phosphate-mediated method (ProFection Mammalian Transfection System; Promega). Viral supernatants were harvested and used to transduce spleen B cells from C57BL/6 mice, as we reported (24, 30, 54), after a 12 h activation by LPS. Transduced B cells were then stimulated by LPS plus TGF-β, IL-4, IL-5, RA and anti-Igδ mAb for 96 h before analyzing GFP^+^ B cells for surface IgA expression as well as intracellular AID, Smad2, Smad3 and Smad4 expression.

### Statistical Analyses

All statistical analyses were performed using Excel (Microsoft) or GraphPad Prism^®^ software. Differences in Ig titers, CSR and RNA transcript expression were analyzed by Student’s paired (*in vitro*) and unpaired (*in vivo*) *t*-test assuming two-tailed distributions.

## Results

### miR-146a Deficient Mice Show High IgA Levels, Increased IgA^+^ B Cells in Multiple Body Districts and Kidney IgA Deposition

**Figure 1:**
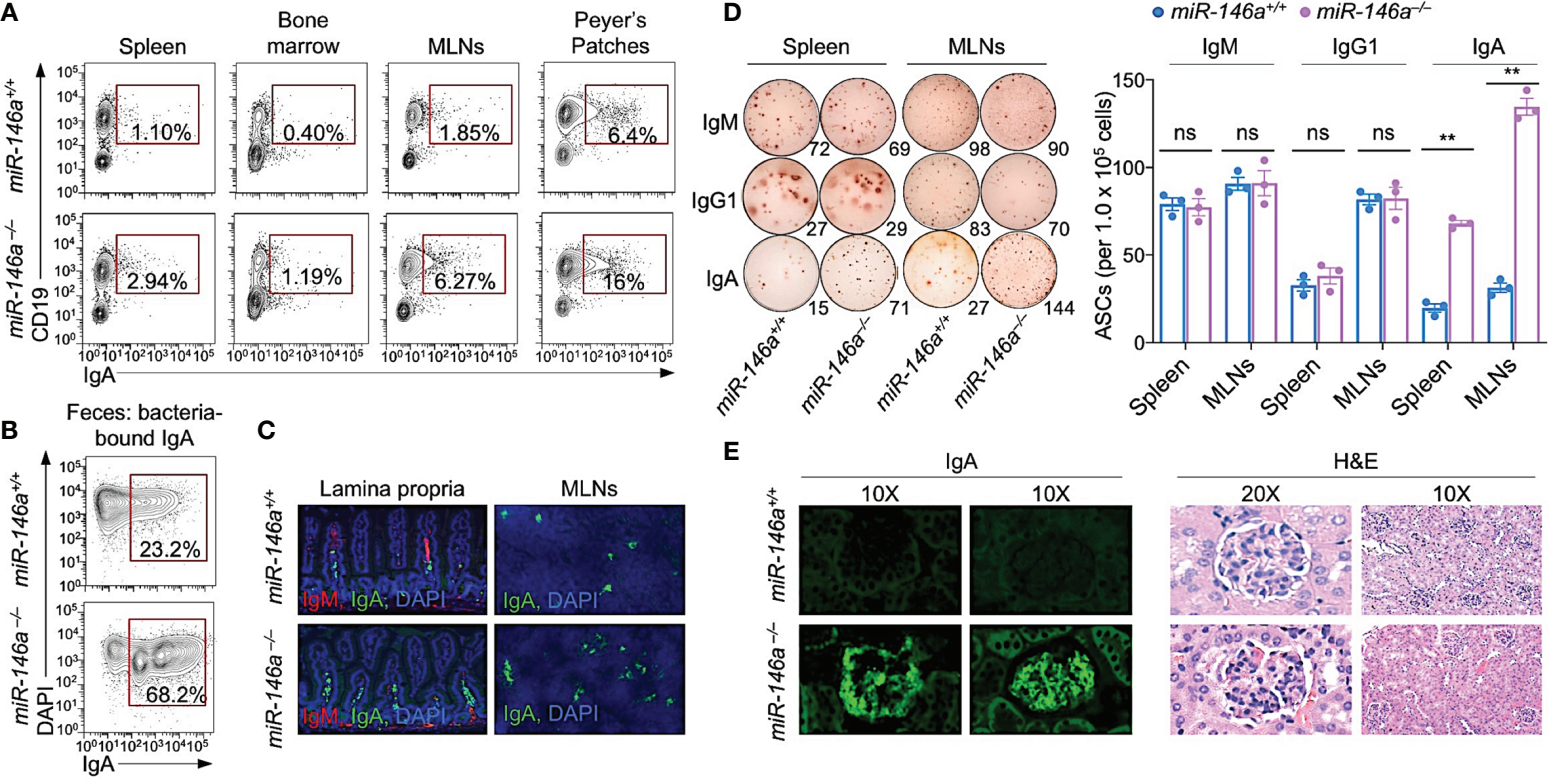
*miR-146a* ^–/–^ mice (12 wk of age) displayed elevated systemic and gut IgA^+^ B cells, IgA-secreting cells, and kidney IgA deposition, as hallmark of IgA nephropathy. **(A)** IgA^+^ B cells in spleen, bone marrow, mesenteric lymph nodes (MLNs) and Peyer’s patches as analyzed by flow cytometry. **(B)** Fecal bacteria-bound IgA as analyzed by flow cytometry. **(C)** IgM^+^ and IgA^+^ B cells in the lamina propria and MLNs of *miR-146a* and *miR-146a^+/+^* mice as visualized by fluorescence microscopy. **(D)** ELISPOT analysis of IgM-, IgG1- and IgA-secreting cells (ASCs) in spleen and MLNs. **(E)** Photomicrographs of kidney sections from *miR-146a^–/–^* and *miR-146a^+/+^* mice, after fluorescence staining for mouse IgA (left) or hematoxylin and eosin (H&E) staining (right). Data are from one representative of three independent experiments yielding similar results; or mean ± SEM of 3-4 *miR-146a^–/–^* or *miR-146a^+/+^* mice from three independent experiments (**D**, right panel). **p < 0.01, ns, not significant, unpaired *t*-test.

We investigated the impact of miR-146a deficiency on IgA expression and IgA-producing cells in non-intentionally immunized 22-wk-old *miR-146a* ^–/–^ mice. These mice showed higher levels of serum IgA (n = 6, 146.0 ± 10.2 vs. 78.4 ± 16.6 μg eq/ml, *p =* 0.014) and fecal IgA (n = 6, 271.3 ± 134.2 vs. 115.1 ± 28.2 μg eq/ml, *p =* 0.032) as well as fecal bacteria-bound IgA (68.25 vs. 23.2%) as compared to their *miR-146a* ^+/+^ mouse counterparts. *miR-146a* ^–/–^ mice also showed higher frequency of IgA^+^ B cells in spleen, bone marrow, mesenteric lymph nodes (MLNs), intestinal lamina propria and Peyer’s patches (**Figures 1A**–**C**). In addition, they showed a higher frequency of IgA- but not IgM- or IgG-secreting cells in spleen and MLNs (**Figure 1D**) as well as IgA deposition in kidney glomeruli and glomerular sclerosis (**Figure 1E**). Thus, deletion of *miR-146a* increases systemic and local IgA.

### miR-146a Knockout Increases Total and OVA-Specific IgA in Blood and Feces

**Figure 2:**
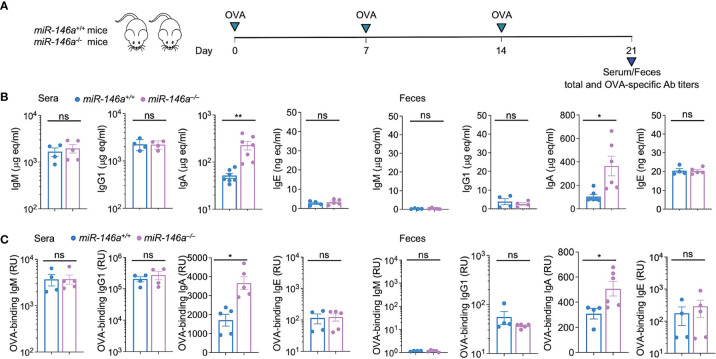
Increased IgA production in *miR-146a^–/–^* mice. **(A)** *miR-146a^–/–^* and *miR-146a^+/+^* mice were administered OVA together with CT *via* intragastric gavage once a week for 3 wks and sacrificed one wk after the last OVA administration for analysis of antibodies, B cells and ASCs. Total **(B)** and OVA-binding **(C)** IgM, IgG1, IgA and IgE titers, as measured by ELISA in serum and feces. **p < 0.01, *p < 0.05, ns, not significant, unpaired *t*-test. Data are mean ± SEM of 4-7 *miR-146a^–/–^* or *miR-146a^+/+^* mice from three independent experiments.

To define the role of miR-146a in the modulation of an antigen-specific response, *miR-146a^+/+^* and *miR-146a^–/–^* mice were administered OVA *via* intragastric route once a week for three consecutive weeks. Serum and feces were collected from each mouse one week after the third OVA administration for analysis of systemic and fecal total and OVA-specific antibody titers, at which time mice were sacrificed for all other studies. *miR-146a^–/–^* mice displayed significantly higher total and OVA-specific IgA (*p* < 0.05) in serum and feces than their *miR-146a^+/+^* counterparts (**Figures 2A**–**C**). By contrast, *miR-146a^–/–^* mice showed no significant changes in total or OVA-specific IgM, IgG1 or IgE titers in serum or feces. Thus, miR-146a deletion increases total and antigen-specific IgA in the circulation and the gut.

### Increased CSR to IgA in Intrinsic B Cell miR-146a Deficient (*µMT/miR-146a^–/–^*) Chimeric Mice

**Figure 3:**
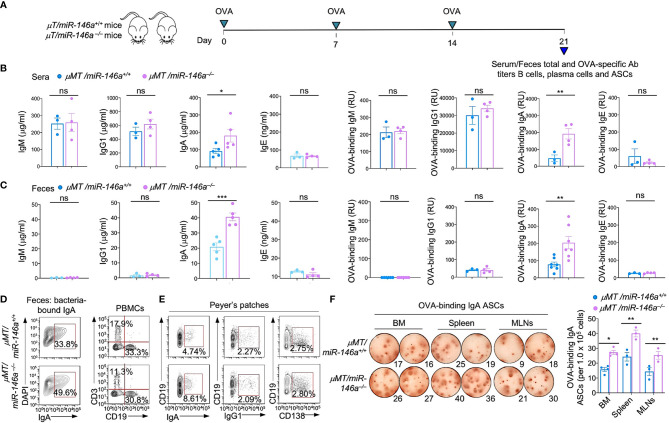
Increased IgA production, IgA^+^ B cells and IgA-ASCs in *μMT/miR-146a^–/–^* chimeric mice. *μMT/miR-146a^–/–^* and *μMT/miR-146a^+/+^* chimeric mice were constructed by mixed bone marrow adaptive transfer. **(A)** All mice were administered OVA together with CT *via* intragastric gavage once a week for 3 wks and sacrificed one wk after the last OVA administration for analysis of antibodies, B cells and ASCs. Total and OVA-binding IgM, IgG1, IgA and IgE titers in sera **(B)** and feces **(C)** as analyzed by ELISAs. **(D)** Fecal bacteria-bound IgA as analyzed by flow cytometry. **(E)** Circulating T (CD3^+^) and B (CD19^+^) cells, Peyer’s patched IgA^+^ B cells, IgG1^+^ B cells and CD138^+^ plasma cells, as analyzed by flow cytometry. **(F)** ELISPOT analysis of OVA-binding IgA ASCs in the bone marrow (BM), spleen and MLNs. ***p < 0.001, **p < 0.01, *p < 0.05, ns, not significant, unpaired *t*-test. Data in **(B, C)** and (**F**, right panel) are mean ± SEM of 3-7 *μMT/miR-146a^–/–^* or *μMT/miR-146a^+/+^* from three independent experiments. Data in **(D, E)** and (**F**, left panel) are from one representative of three independent experiments yielding similar results.

To determine whether the increased IgA production in *miR-146a^–/–^* mice stemmed solely from B cell-intrinsic *miR-146a* deficiency, we constructed *µMT/miR-146a^+/+^* and *µMT/miR-146a^–/–^* chimera mice using myeloablated C57BL/6 mice grafted with (80%) bone marrow cells from *µMT* (B6.129S2-*Ighm^tm1Cgn^*/J) mice specifically deficient in B cells and (20%) purified bone marrow B cells from *µMT/miR-146a^–/–^* mice. Chimeric *µMT/miR-146a^+/+^* and *µMT/miR-146a^–/–^* mice were administered OVA *via* intragastric route once a week for three consecutive weeks. Serum and feces were collected one week after the third administration for systemic and gut titles of total and OVA-specific antibodies analysis, at which time mice were sacrificed (6 wks after bone marrow transplantation) for all other analyses. *µMT/miR-146a^–/–^* mice show significantly elevated systemic and gut IgA (fecal) total and OVA-specific IgA but not IgM, IgG1 or IgE as compared to their *µMT/miR-146a^+/+^* counterparts (**Figures 3A–C**). Accordingly, *µMT/miR-146a^–/–^* mice showed significantly greater amounts of bacteria-bound IgA in feces, greater proportions of IgA^+^ B cells in peripheral blood and Peyer’s patches as well as higher numbers of OVA-specific ASCs in bone marrow, spleen and MLNs with no changes in the proportion of CD19^+^IgG1^+^ B cells or CD138^+^ plasma cells (**Figures 3D–F**). Thus, intrinsic B cell miR-146a deficiency per se underpins increased IgA CSR and increased IgA levels *in vivo*.

### Altered Gut Microbiota in Intrinsic B Cell miR-146a Deficient (*µMT/miR-146a^–/–^*) Chimeric Mice

**Figure 4:**
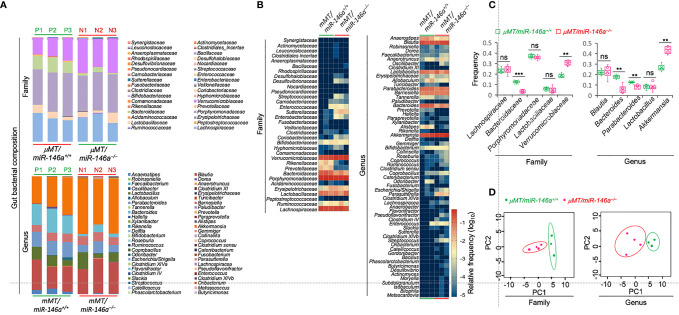
B cell miR-146a deficiency alters gut bacterial microbiome in *μMT/miR-146a^–/–^* mice. Relative abundance of fecal bacteria families and genera **(A, B)** in *μMT/miR-146a^–/–^* and *μMT/miR-146a^+/+^* chimeric mice (*n* = 3 per group) as determined by high-throughput 16s rRNA gene miSeq amplicon sequencing. **(C)** Frequency of five relatively abundant fecal bacteria families and genera in these mice. ***p < 0.001, **p < 0.01, ns, not significant, unpaired *t*-test. **(D)** Principal component analysis of gut bacterial composition of the fecal bacteria families and genera in *μMT/miR-146a^–/–^* and *μMT/miR-146a^+/+^* chimeric mice. Data are from three independent experiments.

As IgA is critical for microbiome homeostasis at the intestinal mucosa and as *miR-146a* deletion resulted in major increases of IgA-producing B cells as well as total and specific IgA, we hypothesized that miR-146a deficiency would impact the composition of gut bacteria, thereby further emphasizing the role of miR-146a in IgA response. We analyzed the fecal microbiota in *µMT/miR-146a* ^–/–^ mice by 16S rRNA gene amplicon sequencing. In all three *µMT/miR-146a* ^–/–^ mice analyzed, the increased IgA production associated with an altered gut bacterial composition as compared to the three *µMT/miR-146a* ^+/+^ mouse counterparts. We found that *Verrucomicrobiaceae, Bacteroidaceae, Porphyromonadaceae* and *Lachnospiraceae*, which are among the most abundant bacterial taxa in feces, were the major families represented in the gut (fecal) microbiome of *µMT/miR-146a* ^+/+^ mice (**Figures 4A**–**C**). *Verrucomicrobiaceae* and particularly its *Akkermansia* genus bacteria have been shown to be physiologically bound by IgA and to positively correlate with IgA level in the gut (59–61). Accordingly, *Verrucomicrobiaceae* and *Akkermansia* bacteria were significantly increased in *µMT/miR-146a* ^–/–^ mice, in which IgA were elevated. Bacteria of the *Bacteroidaceae* family and particularly its *Bacteroides* genus bacteria, which are typically abundant in the colon but not generally bound by IgA (59, 60), were decreased in *µMT/miR-146a* ^–/–^ mice (**Figures 4A**–**C**). Bacteria of the *Clostridia* class, including those of the *Lachnospiraceae* family and the *Blautia* genus, members of the phyla *Firmicutes*, which are also not bound by IgA *in vivo* (59, 60) were not altered in *µMT/miR-146a^–/–^* mice (**Figures 4A**–**C**). *Rikenellaceae* and *Prevotellaceae* family bacteria, which may or may not be bound by IgA, were decreased, while *Sutterellaceae* and *Enterobacteriaceae*, which also may or may not be bound by IgA, were increased in *µMT/miR-146a* ^–/–^ mice. Overall, fecal bacteria families and genera were consistently different in *µMT/miR-146a* ^–/–^ and *µMT/miR-146a* ^+/+^ mice, as revealed by principal component analysis (PCA) (**Figure 4D**). Thus, intrinsic B cell miR-146a deficiency, as in *µMT/miR-146a* ^–/–^ chimeric mice, leads to increased IgA levels and altered composition of the gut microbiota.

### B Cell Intrinsic *miR-146a* Deficiency Increases CSR to IgA

**Figure 5:**
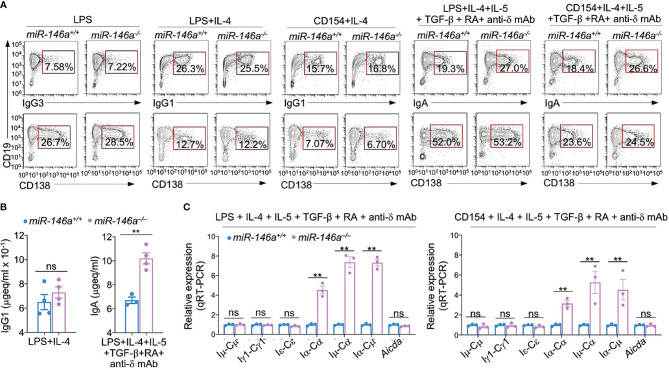
miR-146a ablation results in increased germline *Iα-Cα* transcripts and enhanced CSR to IgA**. (A)** *miR-146a^–/–^* and *miR-146a^+/+^* B cells were stimulated with LPS, LPS or CD154 plus IL-4, LPS or CD154 plus IL-4, IL-5, TGF-β, RA and anti-δ mAb, and cultured for 96 h. IgG1^+^, IgG3^+^ and IgA^+^ B cells and CD138^+^ plasma cells as analyzed by flow cytometry. Data are one representative of three independent experiments yielding similar results. **(B)** IgG1 and IgA concentrations in culture fluids from *146a^–/–^* and *miR-146a^+/+^* B cells stimulated by LPS plus IL-4 or LPS plus IL-4, IL-5, TGF-β, RA and anti-δ mAb, as measured by ELISA. **(C)** *miR-146a^–/–^* and *miR-146a^+/+^* B cells were stimulated by LPS or CD154 plus IL-4, IL-5, TGF-β, anti-δ mAb and RA, and cultured for 72 h. Expression of germline *Iμ-Cμ*, *Iγ1-Cγ1*, *Iα-Cα* and *Iϵ-Cϵ* transcripts, circle *Iα-Cμ* and post-recombination *Iμ-Cα* transcripts, as well as *Aicda* transcripts as analyzed by qRT-PCR and normalized to *β-Actin.* Data are ratios to *miR-146a^+/+^* B cells (set as 1; means ± SEM of three independent experiments). **p < 0.01, ns, not significant, paired *t*-test.

To further define the role of miR-146a in CSR, we stimulated *miR-146a* ^–/–^ and *miR-146a^+/+^* B cells with LPS or CD154 plus IL-4 (to induce CSR to IgG1), LPS alone (CSR to IgG3), LPS or CD154 plus IL-4, IL-5, TGF-β, anti-δ mAb and RA (CSR to IgA). In addition to CSR, all these stimuli also induce plasma cell differentiation, albeit at different degrees. After a 96 h culture, CSR to IgA in *miR-146a* ^–/–^ B cells was significantly increased as compared to *miR-146^+/+^* B cells (27.0% vs 19.3% upon stimulation by LPS plus IL-4, IL-5, TGF-β, anti-δ mAb and RA; 26.6% vs 18.4% upon stimulation with CD154 plus IL-4, IL-5, TGF-β, anti-δ mAb and RA) (**Figure 5A**). Increased CSR to IgA in *miR-146a* ^–/–^ B cells was specific, as these B cells showed no alteration in CSR to IgG1 or differentiation to plasmablasts/plasma cells (as determined by proportion of CD19^+^ IgG^+^ B cells and CD138^+^ cells). The specificity of increased IgA CSR in *miR-146a* ^–/–^ B cells stimulated by LPS or CD154 plus IL-4, IL-5, TGF-β, anti-δ mAb and RA was emphasized by the increased germline *Iα-Cα* transcripts, post-recombination *Iμ-Cα* transcripts, post-recombination circle *Iα-Cμ* transcripts and increased IgA secretion as compared to *miR-146a^+/+^* B cells, as well as by the unchanged IgG1 secretion, and germline *Iγ1-Cγ1* and *Iϵ-Cϵ* transcripts in the very *miR-146a* ^–/–^ B cells (**Figures 5B, C**). Finally, the specific impact of the *miR-146a* on *Igα* locus transcription and recombination was further underlined by the normal level of *Aicda* transcripts in *miR-146a* ^–/–^ B cells undergoing CSR to IgA. Thus, intrinsic B cell *miR-146a* deficiency specifically enhances CSR to IgA while not affecting *Aicda* expression or plasma cell differentiation.

### The Stimuli That Induce CSR to IgA Repress B Cell miR-146a, Thereby Upregulating Smad2, Smad3 and Smad4 Expression

**Figure 6:**
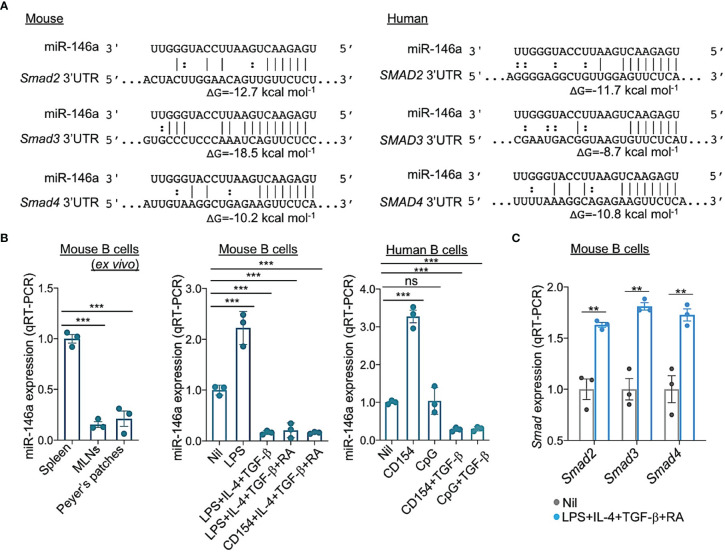
miR-146a targets 3’UTR of *Smad2, Smad3* and *Smad4* mRNAs and is downregulated by the stimuli that induce CSR to IgA in human and mouse B cells. **(A)** Alignment of miR-146a with its target sites in the 3′ UTR of human and mouse *SMAD2/Smad2*, *SMAD3/Smad3* and *SMAD4/Smad4* mRNAs. The complementary base pairing between miRNA-146a and seed sequences within the 3’ UTRs of *SMAD2/Smad2*, *SMAD3/Smad3* and *SMAD4/Smad4*, indicating miR-146a ability to silence *SMAD2/Smad2*, *SMAD3/Smad3* and *SMAD4/Smad4* transcripts. Gibbs free energy (Δ*G*) of nucleic acid folding and hybridization calculated by Mfold. Watson–Crick base-pairing (“|”); wobble base-pairing (“:”). **(B)** Expression of miRNA-146a in mouse spleen, MLN and Peyer’s patch B cells *ex vivo*; and in mouse spleen B cells stimulated for 72 h with Nil, LPS, LPS plus IL-4 and TGF-β, LPS or CD154 plus IL-4, TGF-β and RA, or human B cells stimulated for 72 h with Nil, CD154, CpG, and CD154 or CpG plus TGF-β, as analyzed by qRT-PCR. Values were normalized to expression of small nuclear/nucleolar RNAs Rnu6, Snord61, Snord68, and Snord70, and depicted as relative to the expression of miRNA-146a in spleen B cells stimulated with Nil, set as 1. **(C)** Expression of *Smad2, Smad3* and *Smad4* transcripts in mouse B cells stimulated for 72 h with Nil or LPS plus IL-4, TGF-β and RA, as analyzed by qRT-PCR and normalized to *β-Actin*, and depicted as relative to the expression of these genes in spleen B cells stimulated with nil, set as 1. Data are mean ± SEM from three independent experiments. ***p < 0.001, **p < 0.01, ns, not significant, unpaired *t*-test.

Initiation of CSR to IgA requires TGF-β signaling which induces expression of Smad2, Smad3 and Smad4. Activated Smad2 and Smad3 dimerize and together with Smad4 bind to SBEs in the *Iα* promoter, thereby initiating germline *Iα-Cα* transcription, the first step in CSR to IgA (62). Prompted by luciferase reporter assays showing miR-146a targeting of *SMAD4* mRNA 3’UTR (63–65), we used miRNA-mRNA complementarity prediction tools (TargetScan.org, miRNA.org and miRbase.org) to identify miR-146a-specific target sites in the 3’UTRs of human and mouse *Smad2/SMAD2, Smad3/SMAD3* and *Smad4/SMAD4* (**Figure 6A**), suggesting a potentially silencing of *Smad2, Smad3* and *Smad4* by miR-146a in both human and mouse B cells. This implies that the miR-146a expression level, which is high in resting B cells, must be dampened by those stimuli that induce CSR to IgA to free *Smad2/SMAD2, Smad3/SMAD3* and *Smad4/SMAD4* genes from miR-146a silencing for CSR to IgA to unfold. Toward addressing this postulated miR-146a modulatory function on CSR to IgA, C57BL/6 mouse B cells were stimulated with LPS or CD154 plus IL-4, TGF-β with or without RA, as well as human B cells with CD154 or CpG plus TGF-β to induce CSR to IgA. miR-146a expression was significantly downregulated (*p* < 0.01) in such mouse and human B cells stimulated to undergo CSR to IgA as compared to (mouse) B cells stimulated by nil or (human) B cells stimulated by CD154 or CpG alone (in absence of TGF-β), which does not induce CSR to IgA (**Figure 6B**). The downregulation of *miR-146a* in B cells undergoing CSR to IgA *in vitro* mimicked the downregulation of miR-146a in B cells undergoing IgA CSR *in vivo*, as in MLNs and Peyer’s patches, as opposed to the high level of miR-146a in spleen B cells among which the B cells switching to IgA are a minor proportion of the overall B cell population (in non-intentionally immunized mice) (**Figure 6B**). In mouse B cells induced to undergo CSR to IgA by LPS plus IL-4, TGF-β and RA, miR-146a downregulation was concomitant with increased *Smad2, Smad3* and *Smad4* expression (**Figure 6C**). Thus, the stimuli that induce B cells to undergo CSR to IgA downregulate miR-146a, thereby freeing *Smad2/SMAD2, Smad3/SMAD3* and *Smad4/SMAD4* from miR-146a inhibition and promoting expression of these transcription factors.

### miR-146a Ablation Leads to Further *Smad2, Smad3* and *Smad4* Upregulation in B Cells Induced to Undergo CSR to IgA

**Figure 7:**
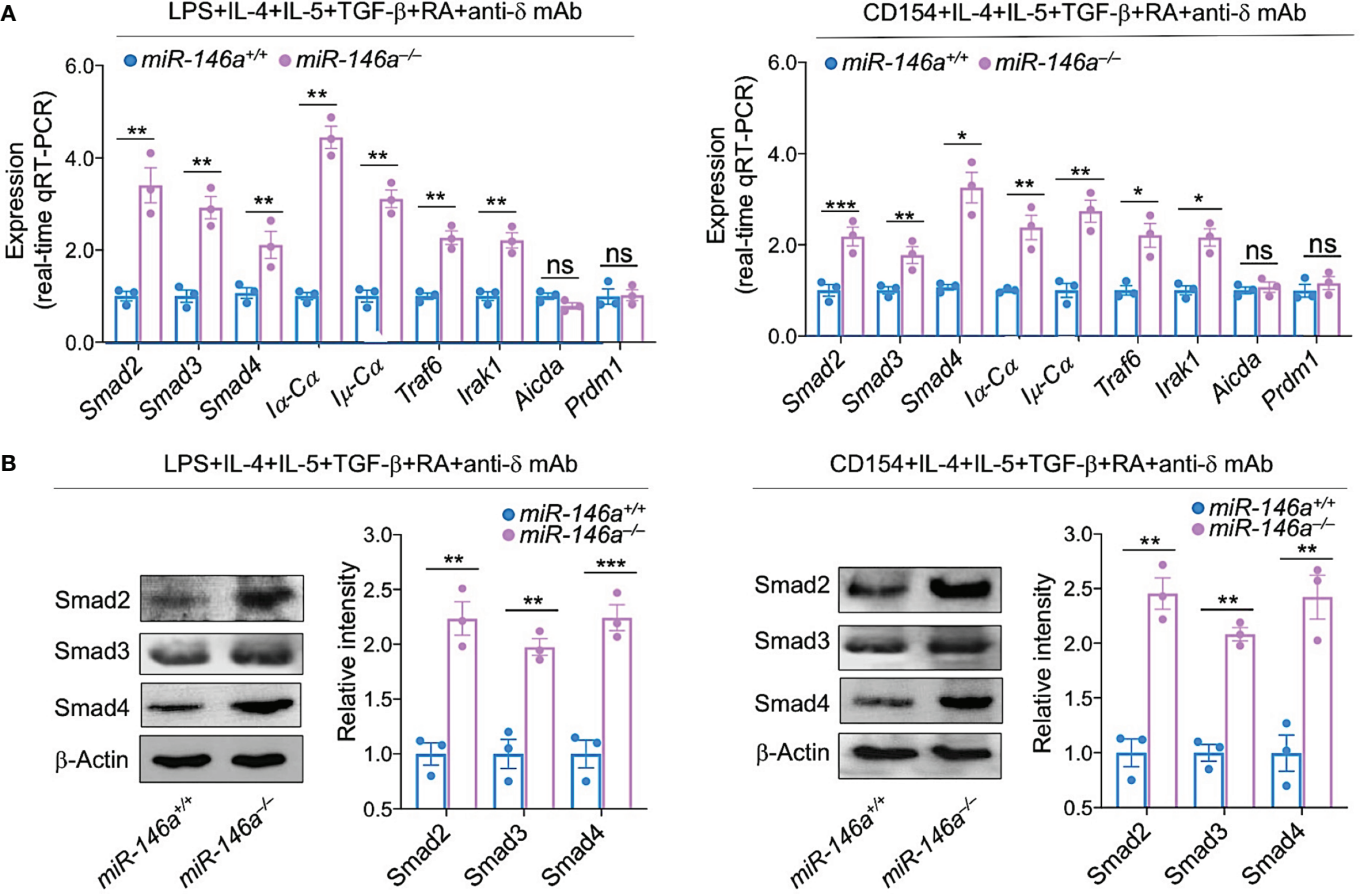
miR-146a deletion increases expression of *Smad2, Smad3* and *Smad4*, germline *Iα-Cα* and post-recombination *Iμ-Cα* transcripts. *miR-146a^–/–^* and *miR-146a^+/+^* B cells were stimulated with LPS or CD154 plus IL-4, IL-5, TGF-β, RA and anti-δ mAb, and cultured for 72 h. **(A)** The expression of *Smad2*, *Smad3* and *Smad4* as well as *Iα-Cα, Iμ-Cα, Traf6, Irak1, Aicda* and *Prdm1* transcripts was analyzed by qRT-PCR performed in triplicate and normalized to *β-Actin.* **(B)** Expression of Smad2, Smad3 and Smad4 proteins as analyzed by immunoblotting. Densitometry quantification of immunoblotting signals was normalized to β-Actin levels and depicted as ratios of readings in *miR-146a^–/–^* and *miR-146a^+/+^* B cells to the average value in *miR-146a^+/+^* B cells. Data are ratios to stimulated *miR-146a^+/+^* B cells (set as 1; means ± SEM of three independent experiments). ***p < 0.001, **p < 0.01, *p < 0.05, ns, not significant, unpaired *t*-test.

Having shown that IgA CSR-inducing stimuli downregulated miR-146a resulting in increased *Smad2, Smad3* and *Smad4* expression, germline *Iα-Cα* transcription, followed by post-recombination *Iμ-Cα*, circle *Iα-Cμ* transcripts, and CSR to IgA, we wanted to prove that ablation of *miR-146a* leads to further *Smad2, Smad3* and *Smad4* expression (critical for *Iα-Cα* transcription and initiation of CSR to IgA). To this end, we stimulated *miR-146a* ^–/–^ B cells and for comparison *miR-146a^+/+^* B cells with IgA CSR-inducing stimuli LPS or CD154 plus IL-4, IL-5, TGF-β, anti-δ mAb and RA. Whether stimulated by LPS or CD154, *miR-146a* ^–/–^ B cells upregulated *Smad2*, *Smad3* and *Smad4* transcripts as well as Smad2, Smad3 and Smad4 proteins at higher levels than their equally stimulated *miR-146a^+/+^* B cell counterparts (**Figures 7A, B**). They also underwent greater CSR to IgA, as indicated by the significantly higher expression of germline *Iα-Cα* and post-recombination *Iμ-Cα* transcripts, as well as *Traf6 a*nd *Irak1*, both genes whose proteins are involved in TGF-β activation signaling pathways (66). miR-146a specificity for Igα locus CSR regulation was further emphasized by the comparable levels of induced *Aicda* and *Prdm1* expression in *miR-146a* ^–/–^ and *miR-146a^+/+^* B cells (**Figures 7A, B**). Thus, ablation of miR-146a significantly increases Smad2, Smad3 and Smad4 expression.

### Increased *Smad2, Smad3* and *Smad4* Expression by miR-146a Deletion Leads to Increased Smad2, Smad3 and Smad4 Recruitment to the *Igα* Locus *Iα* Promoter

**Figure 8:**
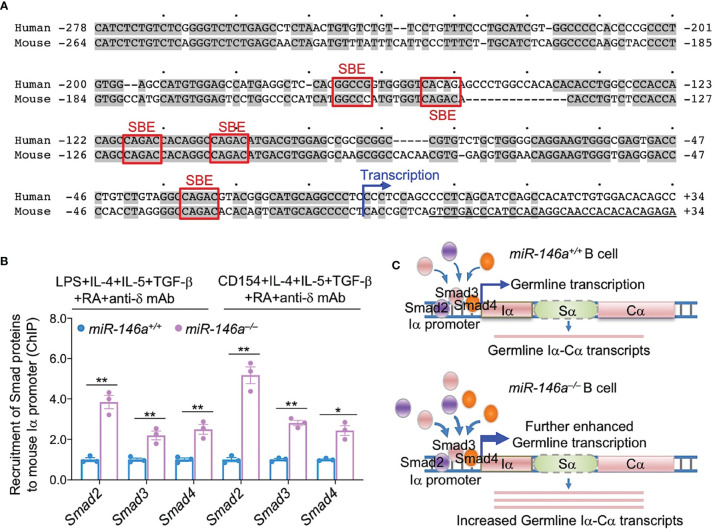
miR-146a deletion results in increased Smad2, Smad3 and Smad4 recruitment to *Iα* promoter. **(A)** Human and mouse *Iα* promoters share five SBEs, three of which are identical and conserved in mouse and human. **(B)** *miR-146a^–/–^* and *miR-146a^+/+^* B cells were stimulated with LPS or CD154 plus IL-4, IL-5, TGF-β, anti-δ mAb and RA for 72 h. The recruitment of Smad2, Smad3 and Smad4 to the *Iα* promoter was analyzed by specific ChIP assays. Data are mean ± SEM from three independent experiments. **p < 0.01, *p < 0.05, unpaired *t*-test. **(C)** Cartoon depicting the increased availability of Smad2, Smad3 and Smad4 in *miR-146a^–/–^* B cells and increased recruitment of these transcription factors to the *IgH* locus *Iα* promoter.

Having shown that induction of CSR to IgA in *miR-146a* ablated B cells leads to even higher levels of Smad2, Smad3 and Smad4, we hypothesized that this would result in increased Smad2, Smad3 and Smad4 recruitment to the *Iα* promoter, a requirement for activation of germline *Iα-Cα* transcription and CSR to IgA. Using TargetScan.org, miRNA.org and miRbase.org, we identified five SBEs [CAGAC and GGC(GC)|(CG) (67)] in the Iα promoter, the DNA region immediately upstream of the *Iα* transcription initiation site. Of such five SBEs, four were CAGAC and one was a GGCCG, overall, highly conserved in mouse and human Iα promoter region (**Figure 8A**). As we hypothesized, ablation of miR-146a, which resulted in significantly increased Smad2, Smad3 and Smad4 levels, led to significantly increased recruitment of Smad2, Smad3 and Smad4 transcription factors to the Iα promoter in B cells activated by LPS or CD154 plus IL-4, IL-5, TGF-β, anti-δ mAb and RA, and as analyzed by specific ChIP (**Figure 8B**). Thus, as freed from miR-146a silencing, increased *Smad2, Smad3* and *Smad4* expression led to significantly increased recruitment of Smad2, Smad3 and Smad4 to the *Iα* promoter SBEs (**Figure 8C**).

### Enforced Expression of B Cell miR-146a Reduces CSR to IgA

**Figure 9:**
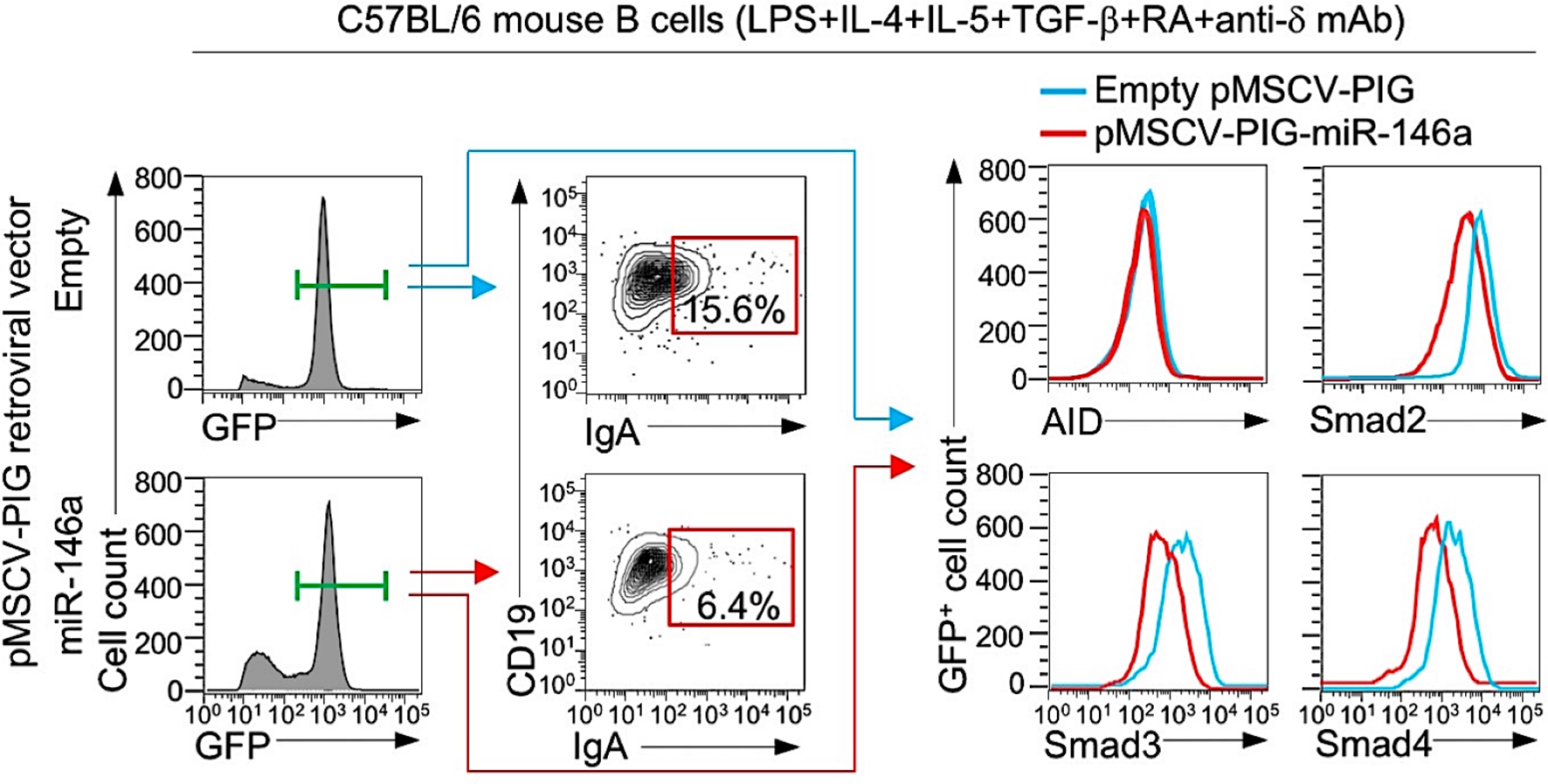
Enforced expression of miR-146a in B cells reduces Smad2, Smad3 and Smad4 expression and CSR to IgA. B cells isolated from C57BL/6 mice were transduced with pMSCV-PIG-miR-146a retroviral vector expressing GFP and miR-146a or empty pMSCV-PIG retroviral vector that expression GFP, then stimulated with LPS plus IL-4, IL-5, TGF-β, anti-δ mAb and RA and cultured for 96 h. Proportions of surface IgA^+^ B cells, and intracellular AID, Smad2, Smad3 and Smad4 levels among the retroviral vector-transduced (B220^+^GFP^+^) B cells were analyzed by flow cytometry. Data are from one representative of three independent experiments.

Having shown that mouse and human B cells physiologically downregulate miR-146a in response to IgA CSR-inducing stimuli to free *Smad2, Smad3* and *Smad4* genes from silencing by this miRNA and, therefore, allowing for greater expression of Smad2, Smad3 and Smad4 and recruitment of these transcription factors to the *Iα* promoter to effect germline *Iα-Cα* transcription, we reasoned that CSR to IgA would be inhibited by high levels of miR-146a. To this end, we enforced expression of miR-146a in LPS-primed B cells by transducing them with retroviral vector pMSCV-PIG-miR-146a (58) expressing GFP and miR-146a or a control pMSCV-PIG vector expressing GFP only. The transduced B cells were subsequently activated by LPS plus IL-4, IL-5, TGF-β, anti-δ mAb and RA, and cultured before being analyzed for expression of IgA, AID, Smad2, Smad3 and Smad4 proteins. The pMSCV-PIG-miR-146a retroviral vector-transduced B cells showed a significant reduction in Smad2, Smad3 and Smad4 expression and CSR to IgA as compared to the pMSCV-PIG retroviral vector-transduced B cells in the face of unchanged AID levels (**Figure 9**). Thus, miR-146a inhibits Smad2, Smad3 and Smad4 expression, thereby dampening CSR to IgA.

## Discussion

IgA is the predominant antibody isotype found in mucous secretions. By maintaining a homeostatic environment at the mucosal surfaces, IgA provides a protective barrier against microbial pathogens and immune evasion. Dysregulation of CSR to IgA, however, can lead to IgA overproduction, such as in hyper IgA syndrome in which IgA kidney deposition leads to immunopathology. While many factors and trans-regulatory elements have been shown to control CSR, there is more to be learned about direct regulation of CSR to IgA, particularly with respect to the role of epigenetics factors, such as non-coding RNAs. Regulation of CSR to IgA must involve Smad2, Smad3 and Smad4. Indeed, these transcription factors provide the critical link between TGF-β signaling and the downstream activation of germline *Iα-Cα* transcription, the initiating event of CSR to IgA. Here we showed that miR-146a plays an important role in the epigenetic modulation of CSR to IgA by inhibiting *Smad2*, *Smad3* and *Smad4* expression. By using *miR-146a* ^–/–^ mice, we showed the lack of miR-146a leads to an enhancement of IgA responses at intestinal and systemic levels. By using mixed bone marrow *μMT/miR-146a* ^–/–^ chimeric mice, we further showed that the modulation of CSR to IgA by miR-146a is B cell-intrinsic. As miR-146a can be downregulated by Smad proteins (50), TGF-β-mediated activation of Smad proteins in B cells would lead to miR-146 reduction, which in turn would result in further expression of Smad proteins themselves. This positive Smads-miR-146a feedback would amplify TGF-β-directed CSR to IgA.

miR-146a would regulate the proliferation of certain immune cells, as suggested by the development of tumors in secondary lymphoid organs and myeloproliferation in aged miR-146a knock-out mice (44). Aberrant miR-146a expression has been identified in autoimmune diseases and disorders, including systemic lupus erythematosus, rheumatoid arthritis, Sjögren’s syndrome and inflammatory bowel disease (68). miR-146a would play a physiological role in cells of the innate and adaptive response (34, 38). It is highly expressed in Tfh and germinal center B cells and thought to restrain the responsiveness of these two immune cell populations (69). miR-146a has been shown to regulate germinal center response by targeting multiple genes associated with CD40 signaling pathway in B cells (39). In aged *CD21-cre miR-146a^fl/fl^* mice, or *CD21-cre miR-146a^fl/fl^* mice immunized (i.p.) with SRBCs or NP-KLH, miR-146a ablation in B cells led to increased germinal center formation and enhanced IgG production. miR-146a has been suggested to restrict the expansion of intestinal T cells and to reduce gut luminal IgA production (45). Indeed, as we showed here, miR-146a expression is lower in gut-associated lymphoid tissues, such as Peyer’s patches and mesenteric lymph nodes, which contain a large portion of IgA-producing cells, than in the spleen which is poorer in IgA-producing cells. *miR-146a* is generally an LPS-responsive gene, as induced by the NF-κB signaling pathway, particularly in innate immune cells, such as granulocytes, monocytes, macrophages and NK cells. Excessive induction miR-146a dampens *Irak1* and *Traf6* (34, 68, 70). *Irak1* and *Traf6* play a significant role in TLR signaling and, possibly, NF-κB activation (71, 72), leading to expression of AID or other genes central to peripheral B cell differentiation. Consistent with a role of miR-146a in targeting *Irak1* and *Traf6* mRNAs, our data showed that expression of *Irak1* and *Traf6* was increased in *miR-146a* ^–/–^ B cells. This, however, did not lead to alteration in *Aicda* or *Prdm1* expression or germline *Iγ1-Cγ1* and *Iϵ-Cϵ* transcripts, further emphasizing that miR-146a directly regulates CSR to IgA but not IgG1 or IgE. The direct modulation of CSR to IgA by miR-146a would provide a novel mechanism in miR-146a-mediated regulation of the antibody response in addition to miRNA146a role in regulation of germinal center formation (39). Upon immunization with OVA *via* intragastric gavage, *miR-146a* ^–/–^ or *μMT/miR-146a* ^–/–^ mice displayed significant increased IgA but not IgG1. In these miR-146a deficient mice, the lack of alteration in IgG1 production was associated with unchanged germline *Iγ1-Cγ1* transcripts and might be due to the type of immunization, which is known to skew the antibody response toward IgA and not to elicit full spleen germinal centers.

TGF-β directs CSR to IgA by activating Smad2 and Smad3 transcription factors, which dimerize to Smad3/Smad3. Activated Smad3/Smad3 dimers recruit Smad4 and in conjunction with co-factors, such as Runx3 and Pu.1, would engage the *Iα* promoter of the *IgH Cα* gene through the five conserved SBEs we identified in this study, thereby inducing germline *Iα-Cα* transcription, the first and critical step leading to CSR to IgA (15, 17–21). Here we showed that miR-146a silences *Smad2, Smad3* and *Smad4* expression in B cells, thereby inhibiting CSR to IgA. We also showed that B cell miR-146a is profoundly downregulated by the stimulus that specifically directs CSR to IgA, i.e., TGF-β, thereby increasing Smad2/Smad3 and Smad4. The specificity of miR-146a downregulation by stimuli that induce Smad proteins and CSR to IgA was emphasized by high levels of miR-146a in human and mouse resting B cells as well as B cells activated by stimuli that are known not to direct CSR to IgA. It was further confirmed by the low levels of miR-146a in B cells undergoing CSR to IgA *in vivo*, as in MLNs and Peyer’s patches, as opposed to the high level of miR-146a in spleen B cells, among which B cells switching to IgA are a minor proportion of the overall B cell population. Besides miR-146a, Smad2, Smad3 and Smad4 may also be targeted by other miRNAs, such as miR-23a, miR-23b, miR-18, miR-27, miR-214 and miR-21 (73). However, virtually all these miRNAs are expressed at low level and are unchanged in B cells undergoing CSR to IgA (our unpublished high throughput miRNA-Seq data), and, therefore, may not play a significant role in the regulation of such CSR.

**Figure 10:**
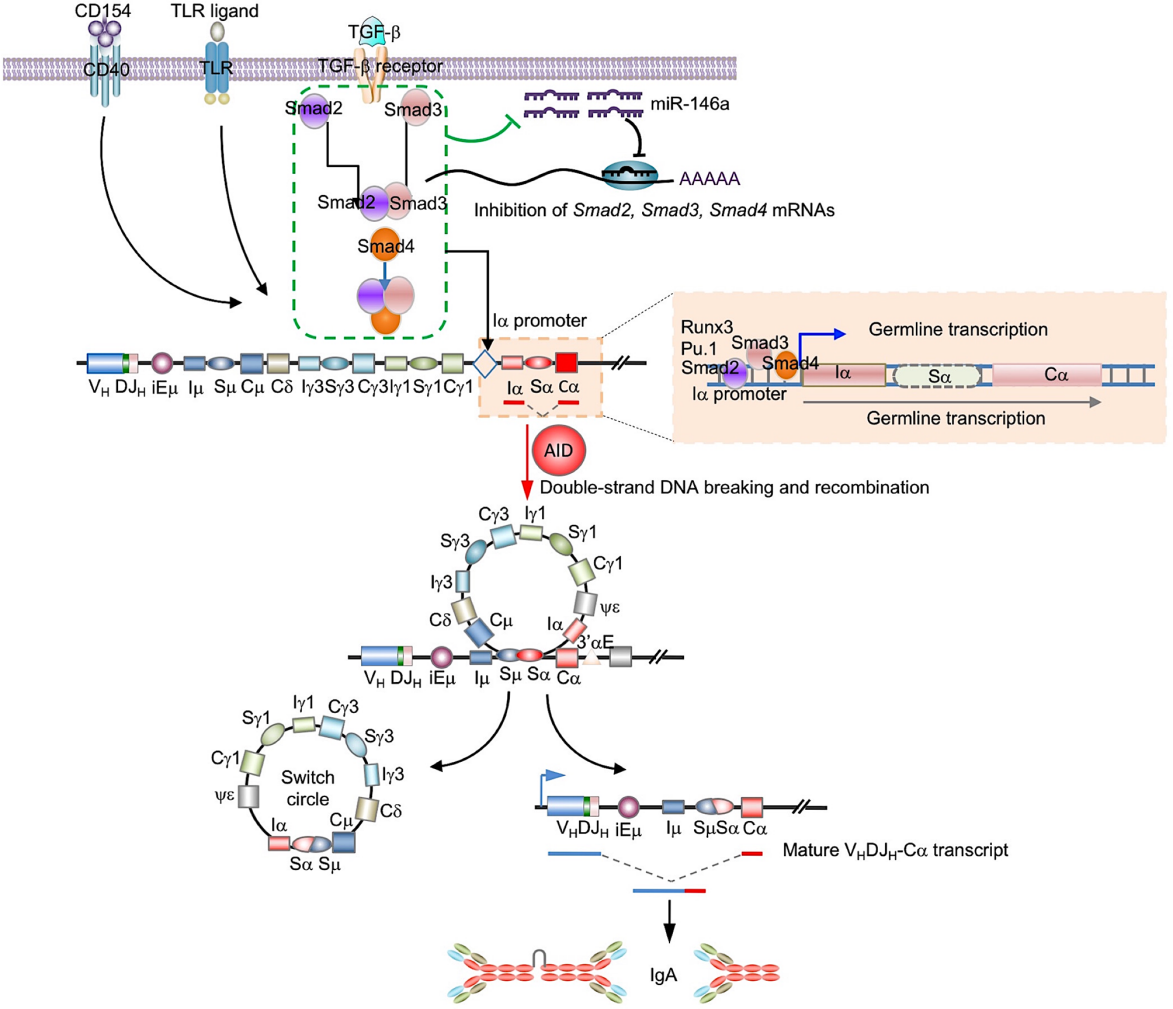
Epigenetic regulation of CSR to IgA by miR-146a. miR-146a targets the 3’UTRs of *Smad2*, *Smad3* and *Smad4* mRNAs to downregulate the expression of Smad2, Smad3 and Smad4, and reduce germline *Iα-Cα* transcription leading to a decreased CSR to IgA and IgA production. In B cells stimulation by TGF-β, activated Smad proteins lead to miR-146 reduction, which results in enhancement of expression of Smad proteins themselves. This miR-146a/Smad loop can amplify TGF-β-induced Smads promotion of CSR to IgA.

The mechanism underlying TGF-β-mediated downregulation of miR-146a is not clear. We suggest here that such downregulation is effected by Smad proteins. These have been shown to modulate miRNA expression through both transcriptional and post-transcriptional mechanisms (74–76), and overexpression of Smad3 and Samd4 has been reported to suppress miR-146 (50). We would argue that in B cells undergoing CSR to IgA, Smad2/Smad3 and Smad4 suppression of miR-146 levels frees *Smad2, Smad3* and *Smad4* from this miRNA-mediated gene silencing, thereby further increasing Smad2, Smad3 and Smad4 expression. This would create an actively self-potentiating Smads-miR-146a loop starting with TGF-β-induction of activated Smads, Smads-mediated miR-146 repression, which results in increased Smad expression, leading to further miR-146a repression, increased Smad expression, and further potentiation of germline *Iα-Cα* transcription and CSR to IgA (**Figure 10**). This actively self-potentiating Smads-miR-146a loop would not rule out the possibility that repression of miR-146a in B cells induced to undergo CSR to IgA may also occur independently of Smad proteins, perhaps through “non-canonical” TGF-β signaling factors such as ERK, p38, RhoA and phosphoinositide 3 kinase (PI3K), which can also be activated by TGF-β (66, 77, 78). Smad-mediated and Smad-independent pathways of repression of miR-146 would not be mutually exclusive, while both dependent on and initiated by TGF-β signaling.

In the intestinal lumen, secretory IgA exist in dimeric form. Dimeric IgA bind to and ‘coat’ commensal bacteria, thereby playing a critical role in shaping the composition and functions of the gut microbiome (79). IgA can limit the access of bacteria to intestinal epithelial cells, facilitate bacterial clearance, regulate bacterial colonization and neutralize disease-associated bacteria (80). Humans and mice lacking IgA display gut microbiota dysbiosis, including reduced overall microbial diversity, altered bacterial composition and increased bacterial translocation (60, 81–84). The role of IgA in shaping the gut microbiota was originally thought to be limited to restriction of growth of specific organisms. Recent data, however, have suggested that IgA can also promote colonization and expansion of select bacterial species (85, 86). We show here that B cell-intrinsic miR-146a ablation, as in *µMT/miR-146a^–/–^* mice, led to increased production of systemic and intestinal IgA including bacteria-bound IgA (in feces), as compared to *µMT/miR-146a^+/+^* mice, thereby resulting in a skwesed selection of gut microbial populations in the absence of miR-146a. *Verrucomicrobiae* class bacteria, in particularly, those of the *Akkermansia* genus, are common members of the human intestinal microbiota and were significantly increased in *µMT/miR-146a^–/–^* mice, in which IgA were elevated. Indeed, such bacteria and have been shown to be positively correlate with IgA level and to be highly coated with IgA (59–61, 83). However, *Bacteroidaceae* family bacteria, particularly those of the *Bacteroides* genus, which, in contrast with *Akkermansia* bacteria, are not generally bound by IgA and are typically abundant in the colon (59, 60), were also decreased in *µMT/miR-146a* ^–/–^ mice – some *Bacteroides* species have been shown to effectively induce production of gut IgA (87), which in turn, could possibly create a negative feedback to the very IgA-inducing bacteria. Similarly, the class *Clostridia* bacteria, including those of the *Lachnospiraceae* family and the *Blautia* genus, members of the phyla *Firmicutes*, which are also not bound by IgA antibodies *in vivo* (59, 60), were also not altered in *µMT/miR-146a^–/–^* mice. Thus, through regulation of IgA levels, B cell-intrinsic miR-146a can play an important role in modulating the steady state of the gut microbiome.

Dysregulated IgA levels and antibody responses are often associated with disease (88). IgA deficiency can lead to severe respiratory tract infections or increase the risk of adverse reactions to blood products (89). Abnormally high IgA levels may cause immune disorders, such as chronic rheumatic disease or chronic gastrointestinal inflammation (88). Here, we demonstrated that miR-146a plays an important role in keeping in check *Smad2, Smad3* and *Smad4* expression and, thereby, inhibiting the TGF-β signaling pathway-dependent induction of CSR to IgA, which results in a significant modulation of the IgA antibody response. Conversely, deletion of miR-146a, as in *miR-146a* ^−/−^ mice, increased systemic and gut IgA, thereby leading to IgA deposition in kidney glomeruli and immunopathology. This is highly evocative of the occurrence of miR-146a single nucleotide polymorphism rs2910164 in childhood hyper IgA syndrome and IgA nephropathy (47). Similarly, high serum IgA levels, as in “hyper IgA” mice, have been shown to result in IgA nephropathy, due to IgA kidney deposition leading to chronic inflammation (90). Interestingly, high IgA levels also occur in most cases of hyperimmunoglobulinemia D syndrome (HIDS), an autosomal recessive disorder characterized by abdominal, articular and skin manifestations in conjunction with recurrent febrile attacks (91). In HIDS patients, high IgA concentrations stem from abnormally high IgA1 levels, which correlate with systemically high IgD levels. While mutations in the mevalonate kinase gene underpin the vast majority of HIDS cases, the root of systemic hyper IgA in HIDS patients remains unknown. By providing a first mechanistic and important insight into epigentic regulation of CSR to IgA expression, our findings open new avenues of investigation of the role of miR-146a-mediated IgA dysregulation in a variety of hyper IgA conditions, such as those in HIDS, “idiopathic” hyper IgA, autoimmune and non-autoimmune inflammatory diseases, cancer and select metabolic disorders.

## Data Availability Statement

16S rRNA gene-sequencing data have been deposited in NCBI Sequence Read Archive (SRA) under BioProject PRJNA777603. All the other data needed to evaluate the conclusions in the paper are present in the paper. Additional data related to this paper may be requested from the corresponding authors.

## Ethics Statement

The animal study was reviewed and approved by The Institutional Animal Care and Use Committees (IACUC) of the University of Texas Health San Antonio.

## Author Contributions

PC conceived and designed the study, planned the experiments, analyzed data, supervised the work, implemented the execution of the overall experimental plan, created figures and wrote the manuscript. SL, GM, CD, DC, and AF, each performed one-two experiments. HZ designed the study, planned the experiments, analyzed data, supervised the work, created figures and wrote the manuscript. All authors contributed to the article and approved the submitted version.

## Funding

This work was supported by NIH grants AI 105813, AI 079705, AI 138944, AI 167416 and the Lupus Research Alliance Target Identification in Lupus Grant ALR 641363 to PC.

## Conflict of Interest

The authors declare that the research was conducted in the absence of any commercial or financial relationships that could be construed as a potential conflict of interest.

## Publisher’s Note

All claims expressed in this article are solely those of the authors and do not necessarily represent those of their affiliated organizations, or those of the publisher, the editors and the reviewers. Any product that may be evaluated in this article, or claim that may be made by its manufacturer, is not guaranteed or endorsed by the publisher.

## Supplementary Material

Supplementary Table 1 PCR primers used in this study.

## Abbreviations

AID, activation-induced cytidine deaminase; ASC, antibody secreting cells; CSR, class switch DNA recombination; IgH, immunoglobulin heavy chain; miRNA, microRNA; OVA, ovalbumin; SBE, Smad-binding element.
