# Supplementary material for: Epigenetic Modulation of Class-Switch DNA Recombination to IgA by miR-146a Through Downregulation of Smad2, Smad3 and Smad4
Source: Front Immunol. 2021 Nov 16;12:761450. doi: 10.3389/fimmu.2021.761450 (PMC8635144; doi:10.3389/fimmu.2021.761450)
Supplement: Supplementary Table 1 — PCR primers used in this study. [file Table_1.pdf]

Supplementary Table 1. PCR primers used in this study.

	Forward primer	Reverse primer
Mouse genes		
Aicda	5’-AGAAAGTCACGCTGGAGACC-3’	5’-CTCCTCTTCACCACGTAGCA-3’
Prdm1	5’-GCTGCTGGGCTGCCTTTGGA-3’	5’-GGAGAGGAGGCCGTTCCCCA-3’
Smad2	5’-GGCCGTCTTCAGGTTTCACAC-3’	5’-CTGGGGTCTCAACTCTCTGGT-3’
Smad3	5’-TGACAAGGTCCTCACCCAGA-3’	5’-CAGGCTGGTGCCTTAGTTGA-3’
Smad4	5’-CGCTCAGCCCTTTAGTGTGT-3’	5’-CTCTTGGTAGAGCTGGCTGG-3’
Irak1	5’-TCCGGAGTTCAAATCCCAGC-3’	5’-TACTTGTGTCCTCTGGGCCT-3’
Traf6	5’-CTACCCGCTTTGACATGGGT-3’	5’-CACCTCTCCCACTGCTTGTT-3’
β-Actin	5’-CTAAGGCCAACCGTGAAAG-3’	5’-ACCAGAGGCATACAGGGACA-3’
Gapdh	5’-TTCACCACCATGGAGAAGGC-3’	5’-GGCATGGACTGTGGTCATGA-3’
Germline transcripts		
Iμ–Cμ	5’-ACCTGGGAATGTATGGTTGTGGCTT-3’	5'-GCAGGCAGGGCTAGATATGG-3'
Iγ1–Cγ1	5'-CTTCCAAGCCAACAGGGCAG-3'	5'-ACCTGTGAGGTGGCTGCGTACTT-3'
Iε–Cε	5'-GACGGGCCACACCATCC-3'	5'-CGGAGGTGGCATTGGAGG-3'
Iα–Cα	5'-GCCATCAAGGCAGGGCCTGGG-3'	5’-TAATCGTGAATCAGGCAG-3’
Post-recombination transcripts		
Iμ– Cα	5’-ACCTGGGAATGTATGGTTGTGGCTT-3’	5’-TAATCGTGAATCAGGCAG-3’
Circle transcript		
Iα–Cμ	5'-GCCATCAAGGCAGGGCCTGGG-3'	5'-GCAGGCAGGGCTAGATATGG-3'
miRNA and other small RNA forward primers (used with Qiagen miScript Universal Primer as reverse primer)		
miR-146a	5’-TGAGAACTGAATTCCATGGGTT-3’	
Snord68	5’-GCTGTACTGACTTGATGA-3’	
Snord70	5’-TTTTGGAACTGAATCTAAGTGATTT-3’	
Rnu6	5’-GCTTCGGCAGCACATATACTAAAAT-3’	
ChIP		
Iα Promoter	5’-CGCATCTCTGTCTCAGGGTC-3’	5’-CTTGCCTCCACGTCATGTCT-3’
